# Simulation-Based System Analysis: Testing Preparedness for Extracorporeal Membrane Oxygenation Cannulation in Pediatric COVID-19 Patients

**DOI:** 10.1097/pq9.0000000000000510

**Published:** 2022-01-21

**Authors:** Alyssa C. Stoner, Robert D. Schremmer, Mikaela A. Miller, Kari L. Davidson, Rachael L. Pedigo, Jamie S. Parson, Christopher S. Kennedy, Eugenia K. Pallotto, Jenna O. Miller

**Affiliations:** * Department of Pediatrics Critical Care Division, Children’s Mercy Hospitals and Clinics, Kansas City, Mo.; † Department of Pediatrics Emergency Medicine Division, Children’s Mercy Hospitals and Clinics, Kansas City, Mo.; ‡ Bioinformatics, Viracor-Eurofins, Lee’s Summit, Mo.; § Extracorporeal Support Department, Children’s Mercy Hospital and Clinics, Kansas City, Mo.; ¶ Center for Pediatric Simulation, Children’s Mercy Hospital and Clinics, Kansas City, Mo.; ** Department of Pediatrics Neonatology Division, Children’s Mercy Hospitals and Clinics, Kansas City, Mo.

## Abstract

**Introduction:**

Coronavirus Disease-2019 presents risk to both patients and medical teams. Staff-intensive, complex procedures such as extracorporeal membrane oxygenation (ECMO) or extracorporeal cardiopulmonary resuscitation (eCPR) may increase chances of exposure and spread. This investigation aimed to rapidly deploy an in situ Simulation-based Clinical Systems Testing (SbCST) framework to identify Latent Safety Threats (LSTs) related to ECMO/eCPR initiation during a pandemic.

**Methods:**

The adapted SbCST framework tested systems related to ECMO/eCPR initiation in the Neonatal and Pediatric Intensive Care Units. Systems were evaluated in six domains (Resources, Processes/Systems, Facilities, Clinical Performance, Infection Control, and Communication). We conducted three high-fidelity simulations with members from the Neonatal Intensive Care Unit General Surgery, Pediatric Intensive Care Unit Cardiovascular Surgery (CV), and Pediatric Intensive Care Unit General Surgery teams. Content experts evaluated systems issues during simulation, and LSTs were identified during debriefing. Data were analyzed for frequency of LSTs and trends in process gaps.

**Results:**

Sixty-six LSTs were identified across three scenarios. Resource issues comprised the largest category (26%), followed by Process/System issues (24%), Infection Control issues (24%), Communication issues (17%), and Facility and Clinical Performance issues (5% each). LSTs informed new team strategies such as the use of a “door/PPE monitor” and “inside/outside” team configuration.

**Conclusions:**

The adapted SbCST framework identified multiple LSTs related to ECMO/eCPR cannulation and infection control guidelines in the setting of Coronavirus Disease-2019. Through SbCSTs, we developed guidelines to conserve PPE and develop optimal workflows to reduce patient/staff exposure in a high-risk procedure. This project may guide other hospitals to adapt SbCSTs strategies to test/adjust rapidly changing guidelines.

## Abstract 2

Supplemental Digital Content is available in the text.

## INTRODUCTION

The emergence of novel coronavirus and associated coronavirus disease (COVID-19) has introduced unprecedented strains on our global health systems.^1^ Efficient person-to-person transmission resulted in over 116 million confirmed cases and over 2.5 million deaths worldwide by early 2021.^2^

Over 5100 patients with COVID-19 have been supported with Extracorporeal Membrane Oxygenation (ECMO) as of early 2021.^3^ The complexity of deploying ECMO in a highly transmissible setting of virulent disease exposes patients and medical staff to potential safety threats.^4^ Simulation is frequently used for crisis response management and emergency preparedness to identify these potential safety threats.^5,6^ Our institution experienced two patients under COVID-19 investigation who required urgent ECMO initiation. The number of essential team members and equipment crowded the patient’s isolation room, and communication was difficult due to the use of personal protective equipment (PPE). The operating room staff wasted supplies due to isolation practices. PPE, already in short supply, was not conserved safely and responsibly. These events demonstrated a need to improve and streamline our internal systems for both patient and staff safety.

The purpose of this safety report is to describe the rapid deployment of the Colman et al’s Simulation-based Clinical Systems Testing (SbCST) framework to develop COVID-19 infection control and emergency preparedness institutional guidelines.^7^ This methodology identified Latent Safety Threats (LSTs) during ECMO/eCPR cannulation and facilitated recommendations for process improvements.

## METHODS

### Context

A request for assistance to rapidly test our process was made to the Center for Pediatric Simulation (CPS). The Center for Pediatric Simulation (CPS) at our institution is composed of multidisciplinary healthcare providers trained in simulation methodology to provide simulation-based training, systems testing, and research for the entire organization. The CPS convened a multidisciplinary key stakeholder group to develop and implement in situ ECMO/eCPR simulations in light of COVID-19 clinical experiences. This group included Pediatric Intensive Care Unit (PICU), Cardiac Intensive Care Unit (CICU) and Neonatal Intensive Care Unit physicians, nursing, respiratory therapists, pharmacy staff, ECMO team, General Surgery (GS) and Cardiovascular Surgery (CVS), Operating Room staff, radiology, and echocardiogram technicians, and Infection Prevention and Control staff.

### Analysis Framework

We utilized the SbCST framework adapted from Colman et al to provide a consistent approach to test clinical processes and human and environmental factors through simulation scenarios. This process is used to detect and report LSTs.^7^ The SbCST approach incorporates peer-review into three phases (development, implementation, and evaluation) to make design decisions in newly built healthcare facilities in the months preceding patient exposure. It utilizes evidence-based safe design principles and usually takes place over months to a year.^7^ This process utilizes the following standardized tools: scenario facilitator guides, observation tools to document LSTs, and Failure Modes and Effects Analysis reports that synthesize LST findings recorded during scenarios.

This approach was condensed over several weeks to facilitate the need for urgent testing during the COVID-19 pandemic. The developmental phase was significantly abbreviated, and implementation was rapid. All components of the developmental phase, including stakeholder engagement, needs assessment, process mapping and scenario development were addressed virtually. We adapted the SbCST facilitator guide to provide a standard template during simulation. It includes the scenario overview, timeline, participant roster and roles, equipment and supplies, and testing objectives. The adapted facilitator guide provided a template for planning, documenting, and debriefing. A COVID-19 ECMO/eCPR comprehensive summary, similar to the SbCST Failure Modes and Effects Analysis, was generated after each simulation scenario to address our institutional needs in the domains of Design/Facility, Resource accessibly/workflow efficiency, COVID process/systems, Clinical Performance, Infection Control, and Communication.^7^

### Intervention

#### Simulation

The high-fidelity simulation was executed three times; twice in the PICU (once with the CVS team and once with the GS team) and once in the NICU with the GS team. A standardized pre-brief described the goals of the SbCST and emphasized process evaluation of each scenario. Two authors (RS and AS) with a combined simulation experience of over 15 years co-facilitated and debriefed the simulation. Although scenario content was consistent, each subsequent simulation was modified based on mitigation strategies learned from the observations and debriefing of the previous scenarios.

### Latent Safety Threats Identification and Mitigation

Seven observers representing key stakeholders and infection prevention were positioned both inside and outside the patient room. They utilized the adapted facilitator guide during the simulation to guide the debriefing process and discuss challenges, LSTs, and possible mitigation strategies. We used Promoting Excellence and Reflective Learning in Simulation and Advocacy Inquiry debriefing frameworks as adjuncts to chronological debriefing.

A COVID-19 comprehensive summary report for each simulation categorized the LSTs as high or low priority, as determined by key stakeholders based on the frequency of observation and level of safety threat to staff. Simulation staff revised the pre-briefing for each subsequent simulation to include the revised mitigation steps for the high-priority LSTs. Final mitigation strategies were incorporated into our institutional recommendations for the facility’s standard COVID-19 guidelines for ECMO/eCPR cannulations.

### Statistical Analysis

Frequency and percentages of each LST domain type were calculated for each scenario to uncover the most prevalent system issues. Analysis was performed in R, version 3.5.^8^

## RESULTS

Three simulation scenarios and debriefings were completed in April 2020, integrating approximately 60 participants and seven observers. Twenty-three participants completed optional evaluations. The application of the SbCST framework identified 66 LSTs during these scenarios (Table 1). Resource (26%) followed by Process/System and Infection Control (24% and 24%, respectively) domains were reported most frequently. Facility and Clinical Performance domain issues were the least frequent (5% and 5%, respectively). Table 2 provides a detailed description of the most common LSTs and mitigation strategies. These strategies were integrated and further tested during subsequent simulations promoting the finalization of hospital guidelines quickly. Participant evaluations agreed this was an acceptable way to test our process.

**Table 1.** Distribution of Latent Safety Threat categories, by Simulation Scenario

Issue Category	CV Surgery, PICU		General Surgery, PICU		General Surgery, NICU		Total	
Resources	5	22%	4	22%	8	32%	17	26%
Process/system	7	30%	3	17%	6	24%	16	24%
Facility	3	13%	0	0%	0	0%	3	5%
Clinical performance	1	4%	2	11%	0	0%	3	5%
Communication	2	9%	4	22%	5	20%	11	17%
Infection control	5	22%	5	28%	6	24%	16	24%
Totals	23		18		25		66	

**Table 2.** Most Commonly Observed LSTs with Associated Recommended Solutions

Latent Safety Threat	Solution	Scenario
*Resource*		
Delayed presentation of support personnel/supplies due to donning PPE	Pre-identify COVID-19 patients eligible for cardiac arrest prevention bundle	CV, GS, NGS
Recording RN had difficulty recording code and communicating with those outside the room	Multiple methods of communication discussed to ease the burden (see below)	GS, NGS
No staff inside the room wearing lead aprons for x-ray exposure after cannulation	Staff options if exposed to x-ray radiation: 1. Stand in bathroom 2. Stand behind portable shield 3. Accept minimal radiation risk	GS, NGS
Bedside RN activated Code blue button but those outside the room did not realize increased acuity of the room	Increased staff awareness that COVID-19 rooms require heightened situational awareness	GS, NGS
*Communication*		
Challenging communication between the team inside the room and outside the room	Assign team member inside and outside room to be communication ambassadors 1. Outside room (code cart manager) 2. Insider room (recorder)	CV, GS, NGS
Communication tools difficult to use and hard to hear with PPE	Two communication methods should be available. Tools developed as below: 1. Dry erase boards: 1 inside and outside room 2. Ascom phones: 1 inside room on speaker, 1 outside room on speaker and muted 3. Bedside landline: on speaker to phone outside room 4. Walkie Talkies: 1 inside and outside room 5. Microsoft Office Teams: 1 inside and outside room via computer, iPad or cell phone	CV, GS, NGS
Medication error due to confusion on which medication was ordered	1. Increased staff awareness of error risk to ensure proper double check 2. Multiple sets of medications should not be passed in at one time	GS, NGS
*Infection Control*		
OR staff not adequately protected entering room without contact/droplet precautions	OR members don contact/droplet precautions into room, then gown again under sterile technique	CV, GS, NGS
Surgeon/assistant without proper eye protection due to loupes	Consider doing cases without loupes, may not be possible in infants	CV, GS
Patients nearby at risk for contamination	Critical event text page sent to instruct RNs to close their patient doors	GS, NGS
All surfaces, equipment and team members immediately outside room are exposed due to multiple door openings and when equipment is removed	1. Door monitor role developed and wears airborne PPE (N95) 2. Equipment that leaves the room (includes carts, tables, x-ray machine, ECMO machine) should be: • Quickly wiped down before leaving the room • Thoroughly cleaned outside the room • Cleaned by staff wearing gown/gloves	CV, GS, NGS
*Clinical Performance*		
Donning and Doffing PPE errors and locations	1. Additional donning/doffing signs placed 2. Use PPE buddy system/PPE monitor	CV, GS

The first scenario identified Resource LSTs with repeated errors in donning and doffing PPE. The lowest Likert score received on the evaluation by participants was related to their ability to use the proper PPE. Confusion surrounding the availability of proper PPE was addressed through the employment of centralized PPE via a bedside cart. PPE super-users (trained nurse monitors donning and doffing of PPE) were integrated during high-risk events. However, contacting the PPE super-users in a timely fashion was later identified as an LST.

We tested and recommended optimal team configuration. Creating the “inside” and “outside” team is a mitigation strategy that crossed multiple LSTs and domains. The design further emphasized the “inside team”, particularly the recording nurse who assumed the communicator role. Subsequent simulations tested the safety and feasibility of this new process and successfully reduced required personnel from 15–17 to 10–12 people. An additional member was added to the “outside team” after the first scenario to mitigate inefficiencies in transferring supplies; decrease multiple, prolonged door openings; and facilitate communication. This “door monitor” is in airborne PPE precautions and decreased door openings by 38% (26–16 occurrences) by the third simulation.

Communication LSTs were prevalent despite various modes of communication. A communicator role for both “inside” and “outside” teams was necessary to facilitate effective communication. Multiple modalities were tested in isolation, but two modalities were ultimately recommended. Another communication LST led to an additional field in the ECMO/eCPR activation page to denote the COVID-19 status of the patient allowing for participants in subsequent scenarios to arrive with proper PPE. The additional information in the ECMO/eCPR paging system decreased confusion for the cannulation teams responding to pages. This system was used 37 times, and 25 patients were ultimately cannulated.

Facility LSTs were difficult to augment; however, recommendations were made to cannulate in the most isolated bed spaces for optimal COVID-19 isolation during ECMO/eCPR cannulation. A specific CVS COVID-19 surgical cart was tested to prevent unnecessary wasting of supplies. Through the SbCST process, we learned this cart could be shared between the surgical services. A COVID-19 eCPR surgical checklist and detailed informational email was provided to all appropriate surgical staff. (**See document, Supplemental Digital Content 1,** which shows…http://links.lww.com/PQ9/A346)

We developed multiple job aides to support the clinical staff. These included infographics, PowerPoint presentations, and surgical checklists. An infographic specific to COVID-19 ECMO/eCPR cannulation (**See document, Supplemental Digital Content 1,** which shows…http://links.lww.com/PQ9/A346) delineates staff configurations and PPE protocols.

## DISCUSSION

During the 2014 Ebola virus disease epidemic, multiple health care institutions and the Society for Simulation in Healthcare recognized and leveraged simulation education as a key component in ongoing and future infectious disease outbreaks.^9^ Similarly, our institution utilized SbCST framework to assess preparedness and improve safety for ECMO/eCPR cannulations during the COVID-19 pandemic. This framework’s ability to be utilized in rapid deployment has also been demonstrated in testing alternative care sites capacity and safety during the COVID-19 pandemic.^10^

Through various highly contagious infectious diseases outbreaks including the Ebola virus disease epidemic, it remains evident that rapid deployment of training education is necessary. Furthermore, the protection of healthcare workers remains a top priority.^9^ Within our already established ECMO-simulation program, we could focus on patient and staff safety by utilizing infection prevention experts. Limiting team size was a primary concern, and our work identified strategies to improve this process. The creation of the “inside” and “outside” teams proved feasible and limited the exposure risk to staff.

For frontline team members, these simulations promoted confidence in the system amidst uncertainty while performing high-level clinical tasks during emergent procedures. A specific COVID-19 ECMO/eCPR cannulation infographic available on the organization’s internal website promotes sustainability of the mitigation strategies and accessibility of the process for staff to easily review.

The SbCST simulations demonstrated 66 LSTs that we could quantify and develop mitigation strategies for in a multidisciplinary team during a time of high anxiety and intensity for staff. Resource, Process/System, and Infection Control domains were identified at the highest frequency suggesting the application of the SbCST framework is suitable for rapid testing of emerging hospital guidelines. With this project, we provide a new application of SbCST to rapidly assess and allow frontline providers to test evolving guidelines.

The communication LSTs proved to be the most challenging to address. The communicator role for both “inside” and “outside” teams is recommended to facilitate effective communication. No single model of communication was sufficiently reliable; thus, employment of at least two modes of communication is recommended.

These systems test developed strategies that have been implemented in other high-risk scenarios, particularly the use of the PPE super-users, the “door monitor,” and the various communication strategies with the modified team configuration. Additionally, the COVID-19 status embedded within the ECMO/eCPR cannulation notification page remains active to assure that all staff members responding to the ECMO/eCPR cannulation have the appropriate PPE. Through the SbCST process, the team focused on how to preserve the safety of team members responding to emergent surgical procedures for patients in extremis.

## CONCLUDING STATEMENT

Our team adapted the SbCST framework to perform a complex system test, identify LSTs and develop solutions to improve patient and staff safety during ECMO utilization during the COVID-19 pandemic. This project demonstrates the value of utilizing an adapted framework to rapidly improve team systems for complex clinical events such as ECMO/eCPR cannulation.

## DISCLOSURE

The authors have no financial interest to declare in relation to the content of this article.

## ACKNOWLEDGMENTS

The authors acknowledge those who made this endeavor possible, including Marita Thompson, MD, Medical Director of Pediatric ECMO and Jeremy Affolter, MD, for their ECMO and resuscitation expertise. Jessica Brunkhorst, MD, Neonatal Simulation Director and Kasey Davis, MD Associate Medical Director of Simulation, Texas Children’s Hospital – Woodlands for their medial simulation expertise. Jessica Rindels, RN infection prevention liaison, for her infection prevention and control insight. The Children’s Mercy Hospital, Kansas City, Missouri, Pediatric Institutional Review Board reviewed this project and determined it did not meet the definition of research involving human subjects.

## Supplementary Material


