# Supplementary material for: Simulation-Based System Analysis: Testing Preparedness for Extracorporeal Membrane Oxygenation Cannulation in Pediatric COVID-19 Patients
Source: Pediatr Qual Saf. 2022 Jan 21;7(1):e510. doi: 10.1097/pq9.0000000000000510 (PMC8782104; doi:10.1097/pq9.0000000000000510)
Supplement: Supplementary file 1 [file pqs-7-e510-s001.pdf]

Supplement 1

Special Procedure for ECMO Cannulation in COVID Rule Out or COVID Confirmed Patients

Pre-Procedure

Team Plan

Inside Room: Cannulating surgeon (& Fellow), OR support staff, Intensivist & Fellow, Bedside nurse (medication administration), Code recorder & communicator, ECMO primer(s), ECMO specialist, 2x Sterile compressors (fellow or nurse), RT, CV Anesthesia (as applicable)

Outside Room: ECMO 2 (gopher), Resource Nurse/RT, CA, PPE & Door monitors, Pharmacist, Order Entry provider

Procedural Items

Equipment:
- Reference COVID equipment check list
- PPE cart for emergency use only

Medication Plan:
- Fentanyl: 10mcg/kg
- Vecuronium: 0.1mg/kg
- Cannulation Heparin 75 Units/kg
- Volume PRN

Don PPE

PPE-Enhanced Contact Droplet Precautions
- Use PPE Buddy system
- PPE guides posted throughout unit
- Remove all jewelry
- Gown
- Gloves
- Eye Protection
- N95/PAPR

Cannulation

Pre-Procedure

- Time out performed
- Leave pump outside room until 100% certain moving forward with cannulation per surgeon
- Discuss cannula sizes
- Hand up to sterile field once ready

Cannulation Tips

Patient Positioning

-Beds 1-4 only have a head board with necessary O2 and air ports

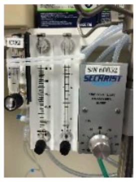

Procedure

Minimize Environmental Noise

1. Close doors off to rest of unit if in PICU rooms 1 – 4
2. Keep cannulation and OR carts away from door opening or ante room for AGP protection – avoid opening doors
3. Speak loudly if PAPRs in use
4. Only bring minimal cannula supplies in room; have gopher grab additional supplies PRN

Post Procedure

Doff PPE

- High Risk for self-contamination
- Refer to PPE conservation/reuse guidelines
- Buddy system
- Don gown to wipe down large machines (ECHO, CXR machine, OR carts)

Additional Considerations

- Consider alternating ECMO specialists Q4-6 hours to achieve breaks from PPE
- Obtain imaging for cannula placement per provider discretion, noting risk of radiographic exposure without donning lead & limited space for machines
- Difficult to hear verbal instructions and orders in full PPE: speak clearly and use closed loop communication
- N95 seals should be checked prior to entering the room and adjust as necessary

Adapted by Jenna Miller and Alyssa Stoner for Critical Care Medicine Mar 26, 2020
Modified by Kari Davidson for ECMO April 13, 2020

4/27/2020 GE

COVID ECPR (General Surgery)

Items Needed:

- COVID ECPR Table (located in CVOR hallway)
- Mayo (optional)
- ECMO tray
- Surgeon loupes and light box/traveling headlight & box
- General travel cart
- 2 Walkie Talkies or Ascom Phones
- OR RN's N95 Masks and Face Shield
- Extra disposable bouffant caps
- Clean, empty case cart
- General ECPR cart and Pack (located outside PICU 39)

Inside Room:

- COVID ECPR Table
- Mayo (optional)
- ECMO tray
- Surgeon preference bag (bottom drawer ECPR cart)
- Pack

Outside Room:

- General travel cart
- General ECPR cart
- Clean, empty case cart

Staffing: (3 RNs total)

- Inside room: 1 Circulator (#1) /1 Scrub RN
- Outside room: 1 Circulator (#2)

PPE Steps:

1. Don COVID PPE (Yellow gown > N95 > Shield > Gray exam gloves)
2. Set up in patient room
3. Remove gray exam gloves
4. Apply Sterillium from wall
5. Don Sterile gown/gloves (over yellow gown)

COVID ECPR (General Surgery)

Front Desk Resource

This process is ONLY for r/o COVID or COVID+ patients!

How do I know the COVID status of the patient?

- COVID status will be communicated in the ECPR page sent to the Trauma Pager
- When calling in General Scrub, let them know COVID status
    - Ask if they have a N95 mask/shield in their locker or if you need to get one for them from COVID cart

- Here are how the various pages will now appear:
    - ECPR Cardiac Surgery NOW IN 11. Covid Positive. MR# 1111111 Patient Wt 2 kg. CALL 55555 to let us know you are coming
    - ECPR General Surgery NOW IN 11. Covid Negative. MR# 1111111 Patient Wt 2 kg. CALL 55555 to let us know you are coming
    - ECMO CARDIAC 11, Covid Pending, Pt Name: Test, MR#: 1111111, Wt: 2 kg. test message, CALL 55555 to let us know you are coming
    - ECMO GENERAL 11, Covid Positive, Pt Name: Test, MR#: 1111111, Wt: 2 kg. Anticipated incision at test. test message, CALL 55555 to let us know you are coming

Items Needed:

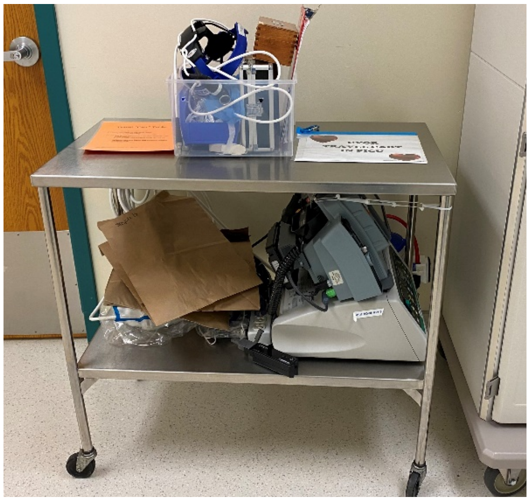

- COVID ECPR Table (located in CVOR hallway where old CVOR travel cart was)
    - CVOR created this table and is graciously allowing us to use this for <u>COVID</u> ECPRs
    - Small back table has Bovie, Defibrillator, Suction, 2 white boards, and Pre-Klenz Spray on it
    - Please remove box of CVOR loupes and N95 masks (place on runabout) before pushing to PICU/ICN
    - Wipe down table/bovie and return to CVOR ASAP in case they need it emergently
- Mayo (optional)
    - Can set up on back table if no Mayo available
- ECMO tray
- Surgeon loupes and light box

4/27/2020 GE

    - Surgeons may not wear a face shield if they wear their loupes due to decrease in accurate visibility
- Traveling light box/head light (available)
- General travel cart
- 2 Walkie Talkies or 2 Ascom Phones
    - Cannot open patient room doors, so these alternative means of communication will allow team in room to communicate to circulator outside if additional supplies are needed
- General ECPR cart and Pack (located outside PICU 39)
- Clean, Empty Case Cart
- OR RNs' N95 Masks
- Extra disposable bouffant caps
    - Wear over surgical cap

Inside Room:

Anything brought into room must be cleaned with purple-top wipes or discarded

- COVID ECPR Table
    - Plug in bovie and suction located on bottom of table
- Mayo (optional)
- ECMO tray
- Surgeon preference bag (bottom drawer ECPR cart)
- Pack

Outside Room:

2nd circulator will stand outside with supplies to retrieve any additional items needed during case; Per Infection Control, items must be passed into room by someone wearing COVID PPE (likely PICU RN)

- General travel cart
- General ECPR cart
- Clean, empty case cart

Staffing:

- Inside room: 1 Circulator (#1) /1 Scrub RN [N95 fit-tested]
- Outside room: 1 Circulator (#2)

Steps for **Donning PPE/Scrubbing** in:

This is recommended practice by Infection Control; Donning procedure for COVID PPE is posted on right side of travel cart for quick reference outside room

1. Don COVID PPE (**Yellow** gown > N95 > Shield > **Gray** exam gloves) outside patient room [Circulator #1 and Scrub RN]
2. Enter patient room and open sterile supplies
3. Remove **gray** exam gloves and unhook **yellow** gown from thumbs [Scrub RN]
4. Apply Sterillium using wall pump
5. Don **STERILE gown and gloves** *over* **yellow** gown

Steps for cleaning COVID ECPR Table and handling dirty instruments after ECPR is complete:

Inside Room:

- **Still in PPE**, *Scrub RN* will:
  - Unclamp all instruments and place them back into the ECMO tray
  - Spray all instruments with Pre-Klenz and close ECMO tray casket with lid
  - Remove all sharp items off the field safely and dispose of sharps in designated sharps disposal bin
  - Dispose of all supplies/drapes that cannot be wiped
- **Still in PPE**, *Scrub RN & Circulator #1* will:
  - Use **purple**-top wipes to wipe ALL surfaces of equipment/supplies/tray brought into the room and Circulator #2 to ready case cart
- Then, *Scrub RN & Circulator #1* will:

1. Doff isolation gown and gloves *only*
2. Perform hand hygiene
3. Don gray exam gloves
4. Prepare "clean" equipment/supplies to leave room

Outside Room:

- Circulator #2 will open case cart by the room door so that Scrub RN can slide ECMO tray into the cart and then Circulator #2 will close and latch case cart door and perform hand hygiene
- Circulator #1 and Scrub RN will remove gloves and perform hand hygiene
- *One* RN that still has N95 and face shield on will don a **yellow** isolation gown and gray exam gloves and re-wipe surfaces of equipment/supplies that were in the COVID room, then doff PPE.

Steps for **Doffing PPE** with N95 and Face Shield:

This is recommended practice by Infection Control

1. Remove gown and gloves
2. Perform hand hygiene
3. Don gray exam gloves
4. Grab **purple**-top wipes
5. Remove face shield and wipe off face shield
6. Place face shield into personal PPE bag
7. Doff gray exam gloves
8. Perform hand hygiene
9. Remove N95 mask (try not to touch the outside and inner part of the mask)
10. Place N95 into brown paper sack and into personal PPE bag
11. Perform hand hygiene

Things to consider:

- Overnight RN may want to keep personal N95 and Shield available at desk during shift in case of COVID ECPR (save time by not running to locker)
- PICU Rooms 1, 2, 3, 4 are considered negative pressure rooms
- ICN negative pressure rooms are located on the "old" side of ICN in POD B
